# Structural and Catalytic Roles of the Disulfide Bonds Cys19–Cys154 and Cys134–Cys199 in Trypsin-like Proteases: Evolutionary Insights for Disulfide Bond Acquisition

**DOI:** 10.3390/molecules31020351

**Published:** 2026-01-19

**Authors:** Maiko Minakata, Yuri Murakami, Orika Ashida, Miki Matsuzaki, Kairi Ogawa, Nanako Saeki, Shigeru Shimamoto, Mitsuhiro Miyazawa, Yuji Hidaka, Nana Sakata

**Affiliations:** 1 Faculty of Science and Engineering, Kindai University, 3-4-1 Kowakae, Higashi-Osaka, Osaka 577-8502, Japan; 2 Institute of Agrobiological Sciences, National Agriculture and Food Research Organization, Tsukuba 305-8634, Japan

**Keywords:** denaturation, disulfide, folding, molecular evolution, mutation

## Abstract

Trypsin is one of the most extensively studied enzymes in biochemistry. However, little information is available on the role of the disulfide bonds to establish the correct conformation and enzyme activity during molecular evolution. To obtain this information, two additional disulfide bonds corresponding to those found in human trypsin were individually or simultaneously introduced into the trypsin-like protease cocoonase (*Bombyx mori*), which contains three consensus disulfide bonds, and structural effects were analyzed. Enzyme assays of the mutant proteins revealed that, during molecular evolution, the Cys19–Cys154 bond contributed to improving substrate recognition (*K*_m_), whereas the Cys134–Cys199 bond contributed to enhancing catalytic turnover (*k*_cat_). In addition, the Cys134–Cys199 disulfide bond significantly increased the structural stability, whereas the Cys19–Cys154 disulfide bond promoted a more compact folded ensemble. Interestingly, when both disulfide bridges were introduced together, their effects acted synergistically, yielding the highest catalytic activity toward the substrate BAEE (*k*_cat_/*K*_m_). Taken together, these findings suggest that trypsin-like proteases evolved through a two-step adaptive process: an initial phase in which the catalytic efficiency (*k*_cat_) and structural stability were enhanced, followed by a second phase in which the fold became more compact, thereby improving the overall enzymatic activity.

## 1. Introduction

Trypsin, a major proteolytic enzyme secreted from the pancreas, is one of the first and most extensively studied serine proteases [1,2,3,4,5,6]. Numerous investigations have focused on its three-dimensional structure, catalytic mechanism, substrate specificity, activity regulation, inhibition, and molecular evolution, often in comparison with chymotrypsin, another pancreatic serine protease [1,3,7,8,9,10]. Despite their relatively low sequence identity (20–30%), trypsin and chymotrypsin share a remarkably similar tertiary structure known as the chymotrypsin fold. The catalytic triads (Ser, His, and Asp) are identically arranged in both enzymes, demonstrating the conservation of the catalytic architecture in the serine proteases [1].

This conserved fold is thought to have been preserved through divergent evolution from a common ancestral protease and serves as the essential structural framework for maintaining catalytic activity, even when substrate specificity differs among serine proteases [1,8]. Such structural conservation is supported by the propeptide region and several intramolecular disulfide (SS) bonds that stabilize the mature enzyme structure [11]. Human trypsin contains five disulfide bonds, and the number of disulfide bonds in trypsin-like serine proteases has increased during molecular evolution [12,13]. Therefore, these disulfide bonds are believed to be crucial for both the structural stability and correct folding of the trypsin-like serine protease family [14,15,16].

Trypsin can have up to six disulfide bonds (SS1 (Cys42–Cys58), SS2 (Cys168–Cys182), SS3 (Cys191–Cys220), SS4 (Cys22–C157), SS5 (Cys136–Cys201), and SS6 (Cys127–Cys232)) [1,16,17]. Among them, three core disulfide bridges—Cys42–Cys58, Cys168–Cys182, and Cys191–Cys220—are conserved across bacterial, fungal, and insect trypsin [17]. These bonds form short loops near the catalytic His57 residue and around the substrate-binding pocket, contributing directly to the structural rigidity of the active site [18]. During the evolution of vertebrates, additional disulfide linkages were introduced, expanding the structural network of the trypsin fold. Early chordates such as tunicates (e.g., *Boltenia* and *Bortyllus*) acquired Cys136–Cys201, while the jawless vertebrate lamprey had already gained two further disulfide bridges, Cys22–Cys157 and Cys127–Cys232, thereby completing the canonical vertebrate set [12]. In trypsin-like serine proteases, several conserved disulfide bonds stabilize extended loop regions that maintain the relative arrangement of the N- and C-terminal β-barrel domains [1,4]. Among these, the newly evolved disulfide bonds found in vertebrate trypsin, such as SS4 and SS5, are positioned within these loop frameworks, suggesting that they contribute to structural resilience and coordinated domain behavior.

Kenesi et al. examined the structural and functional roles of individual disulfide bonds by expressing disulfide-deficient trypsinogen mutants in the *Escherichia coli* periplasmic space [17]. Although they succeeded in obtaining limited information on the expression levels and qualitative enzymatic activities of mutants lacking the Cys42–Cys58 (SS1) and Cys191–Cys220 (SS3) bridges, it was still difficult to rigorously evaluate the precise contributions of these disulfide bonds to folding, structural stability, or catalytic efficiency. Thus, despite extensive structural and enzymatic studies on trypsin, little is known about the role of its disulfide bonds in its folding and catalytic functions. Elucidating how newly acquired disulfide bonds during molecular evolution support correct folding, stability, and protease activity is therefore crucial not only for understanding the molecular evolution of trypsin-like proteases but also for rationally redesigning their structure and function.

A major obstacle to investigating the structural roles of these SS bonds has been the non-specific degradation of trypsin during in vitro refolding and activation reactions, which severely interrupts the biochemical analyses, as described above [19]. To overcome this limitation, we employed cocoonase, a trypsin-like serine protease from the silkworm *Bombyx mori*, which naturally possesses the three conserved disulfide bonds common to the trypsin family [16,20,21]. Cocoonase is secreted during eclosion to hydrolyze sericin, the adhesive component of the cocoon [20]. The precursor form prococoonase consists of a 12-residue propeptide and a 226-residue mature enzyme containing three intramolecular SS bonds [20]. Our previous studies established the recombinant expression of cocoonase in *E. coli*, elucidated the folding mechanism of its propeptide region, identified the catalytic residues (His56, Asp99, Ser193), and successfully suppressed non-specific degradation by site-directed mutagenesis [22,23,24,25]. These achievements enabled us to construct a degradation-suppressed cocoonase (CCN′) without undesired proteolysis during the refolding and self-processing reactions, providing a stable model for analyzing the structural roles of disulfide bonds [24].

In the present study, we aimed to investigate how specific disulfide bonds contribute to the folding and enzymatic properties of trypsin-like proteases by introducing the two additional disulfide bonds found in human trypsin—SS4 (Cys22–Cys157) and SS5 (Cys136–Cys201)—into cocoonase. The positions for introducing these linkages were determined by in silico modeling. In addition, we generated single-disulfide and double-disulfide mutant proteins to compare their folding efficiency, enzymatic activity, and thermodynamic stability. Using this approach, we sought to clarify how the number and positioning of disulfide bonds modulate the structural stability and catalytic properties of serine proteases during the molecular evolution of the trypsin family.

## 2. Results and Discussion

### 2.1. Molecular Modeling of Cocoonase Precursor proCCN′ Mutants Incorporating One or Two Disulfide Bonds

**Figure 1:**
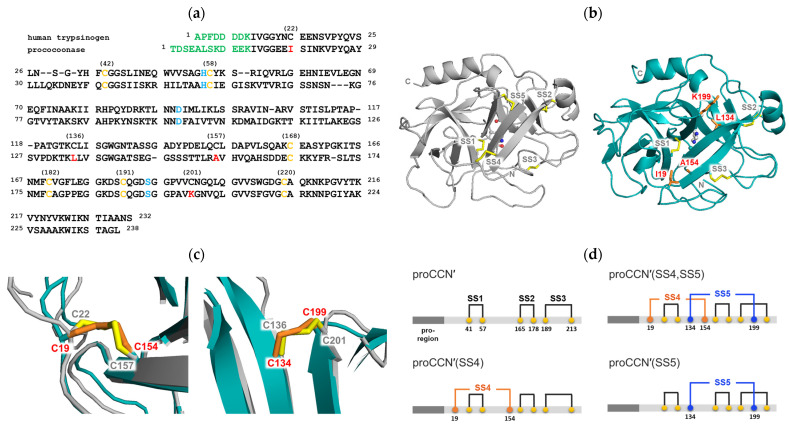
Sequence alignment, structural comparison, and disulfide-bond design of prococoonase. (**a**) Homology alignment of human trypsinogen and prococoonase [13,20,26]. The propeptide region, catalytic residues, and the predicted positions for disulfide bond formation are highlighted in green, sky blue, and red, respectively. The conserved Cys residues are highlighted in yellow. (**b**) Structural models of human trypsin (left, PDB: 1TRN) and cocoonase (right) predicted by AlphaFold2. (**c**) The local structural regions surrounding SS4 (left) and SS5 (right) of human trypsin and the CCN′ mutant proteins containing two predicted disulfide bonds are superimposed. The disulfide bonds in trypsin and the CCN′ mutant proteins are highlighted in yellow and orange, respectively. (**d**) Nomenclature of the recombinant proCCN′ mutants constructed in this study [24].

*Bombyx mori* cocoonase and human trypsin contain three and five disulfide bonds, respectively [13,20,26]. To investigate the structural role of disulfide bonds in the molecular evolution of trypsin-like proteases, we introduced one or two missing disulfide bonds into the cocoonase molecule and examined their effects on its tertiary structure. To predict suitable positions for introducing trypsin-like disulfide bonds, we performed sequence alignment and structural comparison between human trypsin and cocoonase using the EMBOSS Needle alignment program [27]. The results showed 35.2% identity and 49.1% similarity between the two proteins, as shown in Figure 1a. Based on the alignment, two candidate sites, Ile19–Ala154 and Leu134–Lys199, were selected as trypsin-like disulfide bond positions.

Because the three-dimensional structure of prococoonase was unknown, we predicted its structure using AlphaFold2 in this study, as shown in Figure 1b [28], although predicted molecular models had been reported previously for other cocoonases, not for the cocoonase analyzed in the present study [29,30]. The calculated structure was compared with that of human trypsin (PDB: 1TRN), and the predicted mutational positions were superimposed using PyMOL (version 3.0.3), as shown in Figure 1c. The introduced disulfide bonds SS4 (Cys19–Cys154) and SS5 (Cys134–Cys199) in prococoonase corresponded to SS4 (Cys22–Cys157) and SS5 (Cys136–Cys201) of trypsin, respectively. The predicted structure was also analyzed with SSBOND Predict [31] to evaluate the feasibility of introducing additional disulfide bonds. As a result, the predicted sites, SS4 (Cys19–Cys154) and SS5 (Cys134–Cys199), for disulfide bond introduction were confirmed to be appropriate. Indeed, AlphaFold2 predicted the desired structure with the expected disulfide bond pairings between the substituted Cys residues in the designed prococoonase molecules, as shown in Figure 1c. Therefore, these disulfide bonds were introduced into the degradation-suppressed cocoonase, and the recombinant mutant proteins prepared in this study are represented in Figure 1d. Thus, the introduced disulfide bonds were designated as SS4 (Cys19–Cys154) and SS5 (Cys134–Cys199).

### 2.2. Preparation of the proCCN′ and CCN′ Mutant Proteins

All recombinant proCCN′ mutant proteins containing additional disulfide bond(s) were successfully expressed as inclusion bodies in *E. coli* cells using the pET vector system, as described in the Materials and Methods [25]. The denatured proCCN′ mutant proteins were then refolded and purified by cation exchange chromatography, as previously reported [24]. To confirm the formation of disulfide bonds in the mutant proteins, Ellman’s assay was performed on the purified samples, as shown in Figure S1. No free thiol groups were detected, suggesting that all the introduced disulfide bonds had been formed. With respect to the formation of the disulfide bond, its precise pairing could not be determined at this stage. Nevertheless, subsequent enzymatic activity assays, CD, and fluorescence measurements, as described in the following sections, suggested correct disulfide bond formation in the mutant proteins.

To further confirm the recombinant proteins, RP-HPLC analysis was performed, followed by a detailed analysis of the separated peaks using MALDI-TOF/MS, as shown in Figure S2 and summarized in Table S1. The results clearly demonstrated that the obtained proteins corresponded to the intended target proteins.

**Figure 2:**
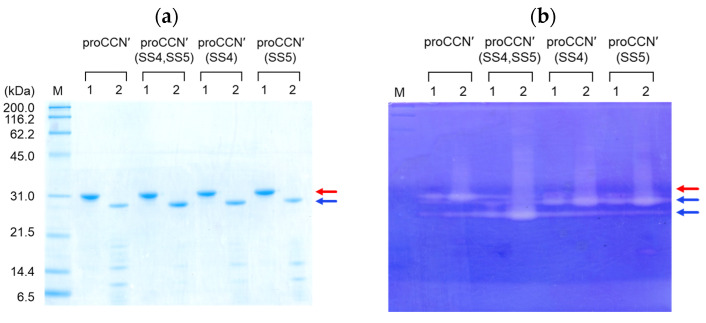
Self-processing of SDS-15%PAGE (**a**) and casein zymography (**b**) of the mutant proteins. Lanes 1 and 2 represent the precursor and mature forms of the CCN′ mutant proteins, respectively. The red and blue arrows represent the precursor and mature forms of the mutant proteins, respectively.

Thus, the designed proteins containing the additional disulfide bond(s) were successfully obtained. Following purification by cation exchange chromatography, the proCCN′ mutant proteins were then subjected to self-processing of the propeptide region for enzyme activation. As expected, all mutant proteins underwent self-processing, as shown in Figure 2a. The resulting mature forms of the CCN′ mutant proteins were then purified by cation exchange chromatography and used as the final purified proteins.

To examine whether the obtained mutant proteins were properly folded, casein zymography, CD, and fluorescence measurements were first conducted. None of the precursor forms exhibited detectable enzymatic activity, as shown in Figure 2b, whereas all purified mature mutant proteins exhibited enzymatic activity in casein zymography, supporting that they had correctly folded into their native conformations. Interestingly, the CCN′(SS4,SS5) mutant protein showed a higher electrophoretic mobility on the casein zymogram (Native-PAGE), suggesting that the introduction of the additional disulfide bonds resulted in a more compact and rigid molecular structure.

**Figure 3:**
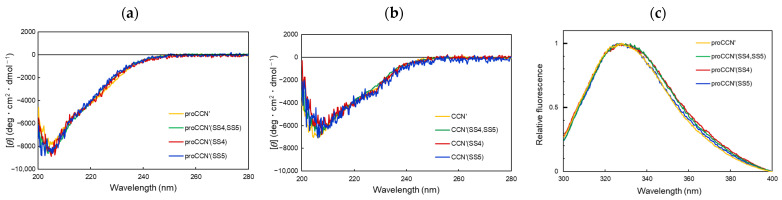
CD spectra and fluorescent spectra of the cocoonase mutant proteins. (**a**) CD spectra of the proCCN′ mutant proteins. (**b**) CD spectra of the mature CCN′ mutant proteins. (**c**) Fluorescence spectra of the proCCN′ mutant proteins. The yellow, green, red, and blue lines represent the CD and fluorescent spectra of the precursor (**a**,**c**) and mature proteins (**b**) of the CCN′, CCN′(SS4,SS5), CCN′(SS4), and CCN′(SS5) proteins, respectively.

To assess whether the introduction of additional disulfide bonds affects the secondary structure, CD measurements were conducted for both the precursor and mature forms of the cocoonase mutants. As shown in Figure 3a, the precursor proteins exhibited nearly superimposable CD spectra, indicating that the added disulfide bonds had little effect on the secondary structure content of the precursor forms. The mature enzymes obtained after self-processing also showed almost identical CD spectra, as shown in Figure 3b. These results revealed that the engineered disulfide bonds preserved the overall secondary structure of both the precursor and mature forms.

This result was also confirmed by intrinsic fluorescence measurements using the Trp residues for comparing the tertiary structures. Specifically, the reference proCCN′ and the mutant proteins with introduced disulfide bond(s) exhibited nearly identical fluorescence spectra, as shown in Figure 3c, indicating that the disulfide bonds had little effect on the tertiary (backbone) structure. These findings demonstrate that the desired mutant proteins were successfully obtained in their correctly folded conformations. These results also indicate that the two disulfide bonds acquired during molecular evolution do not affect the overall folding of trypsin-like proteases.

### 2.3. Protease Activity of the CCN′ Mutant Proteins

To investigate the role of the introduced disulfide bonds in cocoonase on the catalytic activity, the enzymatic activity was measured using BAEE as a substrate, as summarized in Table 1. Interestingly, the enzymatic activity (*k*_cat_/*K*_m_) increased in proportion to the number of introduced disulfide bonds, although all CCN′ mutant proteins exhibited lower catalytic activity than trypsin [32]. These results indicate that disulfide bonds have contributed to the enhancement of specific enzymatic activity during molecular evolution.

The apparent *K*_m_ and the *k*_cat_ values of the mutant proteins are summarized in Table 1. Remarkably, distinct phenomena were observed in the enzymatic activities of the constructed cocoonase mutants. The introduction of additional disulfide bonds (SS4 and SS5) enhanced the catalytic activity more than threefold compared with the reference CCN′ form with three disulfide bonds, although their effects differed. Specifically, the SS4 (Cys19–Cys154) bond decreased the *K*_m_ value, indicating improved substrate recognition, whereas the SS5 (Cys134–Cys199) bond increased the *k*_cat_ value, reflecting enhanced catalytic turnover. Consequently, the CCN′(SS4,SS5) containing both SS4 and SS5 exhibited the highest activity due to the synergistic effects of these two disulfide bonds. These results suggest that, during the molecular evolution toward the human-type trypsin, the increase in the number of disulfide bonds occurred independently to improve the enzymatic performance with respect to both the *K*_m_ and *k*_cat_ values.

### 2.4. Structural Analysis of the proCCN′ Mutant Proteins

**Figure 4:**
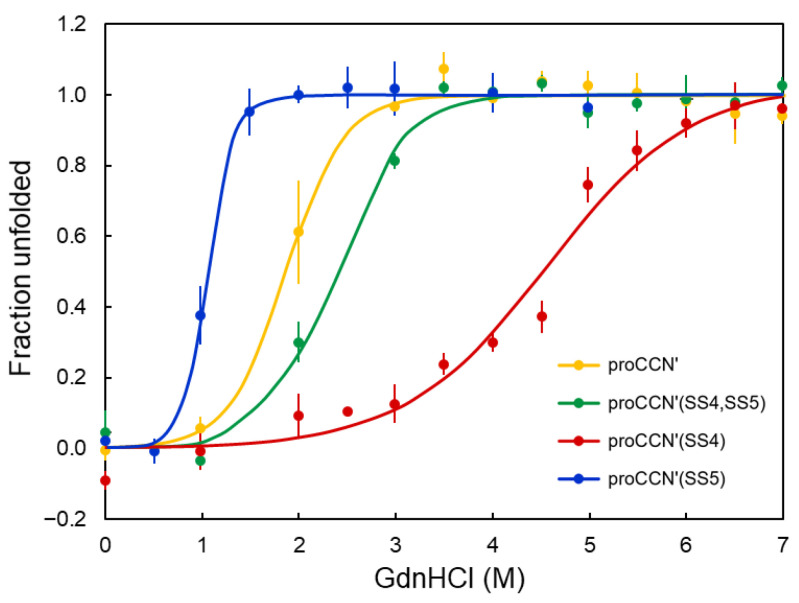
GdnHCl-induced unfolding of the proCCN′ mutant proteins under non-reducing conditions. Fluorescent spectra were recorded in 20 mM sodium phosphate buffer (pH 7.0) containing 0.1 M NaCl at 25 °C. The yellow, green, red, and blue lines represent the unfolding curves of proCCN′, proCCN′(SS4,SS5), proCCN′(SS4), and proCCN′(SS5) proteins, respectively. The experiments were performed in triplicate.

To evaluate the contribution of the engineered disulfide bonds to the structural stability of proCCN′, GdnHCl-induced denaturation experiments were performed under non-reducing conditions using intrinsic fluorescence spectroscopy, as shown in Figure 4. The order of the midpoints of the unfolding transition (*C*_m_) among the mutant proteins was proCCN′(SS4) > proCCN′(SS4,SS5) > proCCN′ > proCCN′(SS5), as summarized in Table 2. In contrast, the unfolding free energy changes (Δ*G*°_N→U_) showed a markedly different trend. Fluorescence measurements revealed a stabilization upon disulfide introduction in the order proCCN′(SS5) > proCCN′(SS4,SS5) ≥ proCCN′(SS4) ≥ proCCN′ (Table 2). Whereas the introduction of SS4 alone or together with SS5 produced only modest increases in Δ*G*° relative to the reference CCN′, the mutant containing only the SS5 disulfide exhibited a substantially larger increase in unfolding free energy.

The introduction of SS5 resulted in a lower *C*_m_ but a higher Δ*G*°, indicating that SS5 enhances the thermodynamic stability of the tertiary structure while increasing the cooperativity of the unfolding transition. This dual effect suggests that SS5 reinforces the hydrophobic core of the β-barrel and promotes a more concerted unfolding process, as described in the next section. Therefore, the increased Δ*G*° represents the dominant contribution of SS5, demonstrating that this disulfide bond plays a stabilizing role within the cocoonase scaffold.

Importantly, *C*_m_ does not directly correspond to the thermodynamic stability (Δ*G*°) but instead reflects how each disulfide bond modulates the unfolding transition [33]. The observed *C*_m_ hierarchy indicates that the SS4 disulfide (Cys19–Cys154) most effectively resists denaturant-induced unfolding, whereas the SS5 disulfide (Cys134–Cys199) shifts the unfolding transition to lower denaturant concentrations, consistent with local structural effects and changes in unfolding cooperativity. The combined proCCN′(SS4,SS5) mutant displayed an intermediate *C*_m_, suggesting that the stabilizing influence of SS4 partially offsets the effect of SS5.

Tryptophan fluorescence further clarified the structural contributions of the two engineered disulfide bonds. The introduction of SS4 increased *C*_m_ without substantially affecting Δ*G*°, consistent with the stabilization of a surface-exposed flexible region that alters the denaturant sensitivity (m-value) rather than the intrinsic energetic cost of unfolding [34]. In contrast, the introduction of SS5 decreased *C*_m_ while increasing Δ*G*°, indicating that SS5 tightens the hydrophobic core, enhances tertiary-structure stability, and increases the cooperativity of unfolding, thereby shifting the midpoint to lower denaturant concentrations.

### 2.5. Role of Disulfide Bonds on the Structural Stability and Enzyme Activity of Trypsin-like Protease During Molecular Evolution

As described in the Introduction, the functional roles of disulfide bonds in trypsin-like serine proteases have remained largely unresolved. To date, only SS3 has been experimentally characterized by Kenesi et al. and Varallyay, E. et al., who reported that the disruption of SS3 affects both the *K*_m_ and *k*_cat_ values, leading to decreases in the substrate recognition ability and catalytic efficiency [17,18]. The roles of the other disulfide bonds, however, have been inferred only from interspecies homology and have not been experimentally verified, especially with respect to molecular evolution.

**Figure 5:**
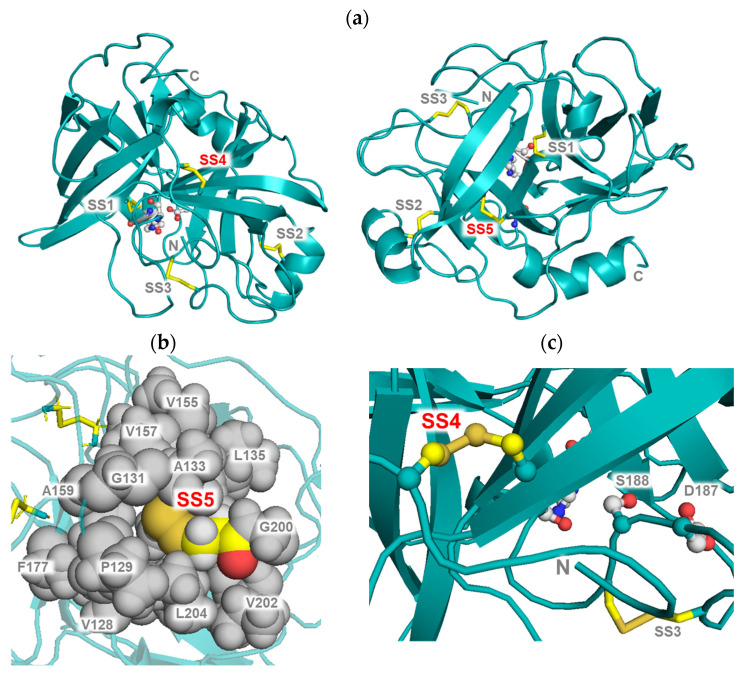
Structural interpretation of the effects of SS4 and SS5. (**a**) AlphaFold2-predicted structures of the CCN′(SS4) (left) and CCN′(SS5) (right) proteins. The incorporated disulfide bonds are highlighted in yellow. (**b**) Local structural comparison of SS5 (Cys134–Cys199). The hydrophobic core region is highlighted in gray. (**c**) Enlarged view of the SS4 region. The introduced SS4 (Cys19–Cys154) links the N-terminal random coil and β-sheet structures.

In the present study, we investigated the contribution of specific disulfide bonds to the molecular structure of the trypsin-like protease family by introducing additional disulfide bonds into cocoonase, as shown in Figure 5a. Our results revealed that the introduction of SS5 thermodynamically stabilized the molecule, as evidenced by the increased free energy required for unfolding. In general, disulfide bonds stabilize proteins by reducing the conformational entropy of the denatured state. In the case of SS5, the Cys134–Cys199 disulfide is positioned within the hydrophobic core that supports the β-barrel forming the base of the substrate-binding pocket (Figure 5b). Introducing this disulfide effectively reinforces the hydrophobic packing in this region, thereby strengthening the tertiary structure and contributing to the observed increase in unfolding free energy.

Interestingly, the SS5 mutant protein exhibited an increased *k*_cat_ value, suggesting that this disulfide bond contributes to enhanced catalytic turnover during molecular evolution. Because the crystal structures of both the wild-type and mutant cocoonase have not yet been determined, the structural basis for the increase in the *k*_cat_ value remains unclear at this stage. However, the pocket-volume analysis at the substrate-binding site using the CASTp program showed that SS5 is located on the opposite side of the β-barrel structure and does not influence the volume or geometry of the substrate-binding pocket, indicating that the observed increase in the *k*_cat_ value cannot be explained by changes in the substrate accommodation, as summarized in Table 3 [35]. Instead, it is possible that SS5 affects the local dynamics or electrostatic environment of the catalytic residues through indirect structural coupling across the β-barrel framework. Similar long-range effects have been reported in other serine proteases, where disulfide bonds or other backbone constraints distant from the active site modulate catalytic turnover by influencing the mobility of the active-site loop, internal strain distribution, or electrostatic preorganization [1,36,37,38]. Although further structural or dynamic analyses are required, the present findings suggest that SS5 may fine-tune the catalytic efficiency of cocoonase through such an indirect mechanism without directly altering the substrate-binding pocket.

Notably, SS5 is highly conserved among trypsin-like serine proteases—present in approximately 78% of reported members [16]. This strong evolutionary conservation suggests that SS5 provides a dual advantage: it enhances catalytic turnover and simultaneously stabilizes the β-barrel core. These combined structural and functional benefits likely contributed to the selective retention of SS5, supporting its role as an essential element for maintaining catalytic preorganization within the trypsin fold.

In contrast, although the introduction of SS4 increased the *C*_m_ value, it had little effect on the unfolding free energy (Δ*G*°). SS4 links the N-terminal random coil and β-sheet structures in the proCCN molecule, restricting the conformation of the N-terminal region of the proCCN molecules, as shown in Figure 5c. In addition, this long-range crosslink likely constrains large-scale motions during unfolding. Such restriction modifies the denaturant sensitivity of the transition and consequently increases *C*_m_, while exerting minimal influence on the intrinsic thermodynamic stability of the fold. Although substrate binding is inherently a dynamic process, when discussed in terms of static structural features, a qualitative relationship between pocket volume and enzymatic activity can be considered. Pocket-volume analysis further revealed that the substrate-binding pocket of the SS4 mutant protein was smaller than that of the reference CCN′ molecule, consistent with the observed decrease in the *K*_m_ value, as summarized in Table 1. This contraction of the binding pocket likely reflects tighter packing of the substrate-recognition loops, leading to stronger substrate interactions [22]. Notably, a similar reduction in the substrate-binding pocket volume was observed in highly active human trypsin (Table 3), suggesting that such structural compaction represents a conserved mechanism for improving substrate recognition in the trypsin family.

Although the present experiments assessed esterase activity using BAEE as a substrate, the results provide insight into the protease activity of trypsin-like enzymes and can be discussed as follows. Taken together, these findings indicate that the two disulfide bonds exert complementary effects on cocoonase. SS5 enhances the catalytic turnover while contributing to higher structural stability, whereas SS4 increases the structural rigidity and improves the substrate affinity. Notably, evolutionary analyses have shown that, in the vertebrate lineage, tunicates first acquired SS5, and the complete disulfide set was later established in lampreys through the subsequent incorporation of SS4 [12]. This evolutionary pattern suggests that SS5 emerged earlier, followed by the later addition of SS4. Therefore, the complementary relationship between SS4 and SS5 suggests that the molecular evolution of trypsin-like serine proteases proceeded through a two-step adaptive process. SS5 was likely acquired first, enhancing both catalytic turnover and structural stability through reinforcement of the β-barrel core. SS4 was incorporated later, increasing the structural rigidity and refining substrate recognition. This stepwise acquisition of disulfide bonds indicates that trypsin-like proteases evolved by progressively optimizing their catalytic function and structural architecture. Although this study focused on the roles of disulfide bonds, during molecular evolution, the increase in the number of disulfide bonds may be accompanied by cooperative interactions with other residues that contribute to the structural scaffold formed by these disulfide bonds [39]. Investigation of such residues will be an important subject of future studies.

## 3. Materials and Methods

### 3.1. Materials

Bz-Arg-OEt (BAEE) was purchased from the Peptide Institute, Inc. (Osaka, Japan). Benzamidine hydrochloride, 2-diisopropylaminoethanethiol hydrochloride (DPAET), and 3,5-dimethoxy-4-hydroxycinnamic acid were purchased from Tokyo Chemical Industry Co., Ltd. (Tokyo, Japan). Urea, 1,4-dithiothreitol (DTT), 5,5′-dithiobis(2-nitrobenzoic acid) (DTNB), oxidized glutathione (GSSG), casein, trifluoroacetic acid (TFA), protein markers for SDS-PAGE, and Tris/HCl were purchased from Nacalai Tesque, Inc. (Kyoto, Japan). The cDNA encoding proCCN′(SS4,SS5) was prepared by Eurofins Japan (Tokyo, Japan) and, its codon bias was optimized for an *E. coli* expression system using the manufacturer’s GENEius software program (http://www.geneius.de/), as shown in Figure S3. All chemicals and solvents used were of reagent grade.

### 3.2. Construction of the Expression Vectors of the Recombinant Mutant Proteins in E. coli Cells

The primer sequences used in this study are summarized in Supplementary Table S2. The cDNAs of the mutant proteins, in which the additional Cys residues were incorporated, were prepared by PCR using the plasmid containing the synthetic cDNA encoding the [K8D,I19C,K63G,K131G,K133A,L134C,A154C,K199C]-proCCN (referred to as proCCN′(SS4,SS5), Figure S3) as the template. The cDNAs encoding the proCCN mutant proteins, [K8D,I19C,A154C]-proCCN′ protein (proCCN′(SS4)) and [K8D,L134C,K199C]-proCCN′ protein (proCCN′(SS5)) were prepared by PCR [25]. The PCR reaction was carried out using Platinum *Pfu* DNA polymerase (Bioneer Co., Ltd., Daejeon, Republic of Korea). The resulting amplified cDNAs were then subcloned into the pET-17b expression vector (Merck KGaA, Darmstadt, Germany), following the introduction of a *Nde*I and an *Xho*I site at its 5′ and 3′ end, respectively. The resulting expression vectors, referred to as pYM4, YM5, and YM6, contained the cDNAs encoding the [K8D,I19C,A154C]-proCCN′ protein (SS4), [K8D,L134C,K199C]-proCCN′ protein (SS5), and [K8D,I19C,L134C,A154C,K199C]-proCCN′ protein (SS4,SS5), respectively. The cDNA sequences of the vectors were confirmed by the Eurofins Japan DNA sequencing service (Tokyo, Japan).

### 3.3. Protein Expression and Purification of the Recombinant proCCN′ Mutant Proteins

The protein expression and crude preparation were performed following a previously reported method [25]. All of the mutant proteins were well expressed and obtained as inclusion bodies in *E. coli* cells and possessed a Met residue at the N-terminus of the proCCN′ mutant proteins, derived from the *Nde*I site during the subcloning. After sonication of the cells, the mixtures were centrifuged at 15,000 rpm for 15 min at 4 °C. The residues were subsequently washed with 0.5% Triton X-100, water, and 80% CH_3_CN/0.05% TFA under sonication. The final products were resuspended in Milli-Q water and the protein levels were estimated by SDS-PAGE analyses using BSA as a standard protein.

### 3.4. Refolding Reaction of the proCCN′ Mutant Proteins

The reduced forms of the recombinant proCCN′ mutant proteins were refolded using a previously described method with minor modifications [24,40]. In brief, the recombinant proteins (ca. 5 mg) were solubilized by treatment with 6 M GdnHCl in 0.2 M Tris/HCl (pH 8.0, 2 mL) containing 20 mM DTT at 50 °C for 30 min. After centrifugation, to remove the DTT, the protein solution was dialyzed twice against 4 M urea/0.05% TFA containing 0.5 M NaCl and the resulting solution was centrifuged at 15,000 rpm for 15 min. The supernatants were mixed with an equal volume of 0.2 M Tris/HCl (pH 8.0) containing 0.5 M NaCl, 10 mM benzamidine, 8 mM DPAET, and 2 mM GSSG and allowed to stand for 2 h at 4 °C. Then, the reaction mixtures were diluted with an equal volume of 0.2 M Tris/HCl (pH 8.0) containing 0.5 M NaCl (the final concentration of urea, 1 M) and 5 mM benzamidine and kept for 2 days at 4 °C. The refolded proCCN′ mutant proteins were dialyzed twice against 20 mM sodium phosphate buffer (100 mL, pH 7.0) containing 20 mM NaCl and 5 mM benzamidine. The resulting solution was subjected to cation exchange chromatography, as described in the following section.

### 3.5. Cation Exchange Chromatography

The proCCN′ and the CCN′ mutant proteins were purified by cation exchange chromatography using the ÄKTA purifier (GE Healthcare Japan, Tokyo, Japan) in the presence of 5 mM benzamidine. Briefly, the protein solutions were loaded onto a HiTrap SP HP column (1 mL, GE healthcare Japan, Tokyo, Japan) pre-equilibrated with buffer A (20 mM phosphate buffer, pH 7.0), and proteins were eluted with buffer B (20 mM phosphate buffer, containing 1 M NaCl, pH 7.0). Protein concentrations in the eluted fractions were determined by Bradford’s method or by UV absorbance, as previously described [22,41].

### 3.6. Self-Processing of the proCCN′ Mutant Proteins

The fractions of the proCCN′ mutant proteins obtained by cation exchange chromatography were transferred into a dialysis membrane (molecular weight cutoff: 12,000–14,000; SEKISUI CHEMICAL Co., Ltd., Osaka, Japan) to remove benzamidine. Dialysis was performed twice (30 min each, 4 °C) against 0.05% aq TFA, followed by three additional dialysis steps (30 min each, 4 °C) against 20 mM phosphate buffer (pH 7.0) containing 0.1 M NaCl. After dialysis, the protein solutions were centrifuged at 12,500 rpm for 15 min at 4 °C, and the supernatant was concentrated using an ultrafiltration device (Amicon Ultra 0.5 mL/30 K; Millipore, Burlington, MA, USA) by centrifugation at 5000× *g* for approximately 5 min at 4 °C, until the protein concentration reached ≥0.8 mg/mL, as determined by UV absorbance [41].

Self-processing reactions were then carried out by incubating the concentrated samples at 37 °C for 30 min followed by 25 °C for 16 h [24]. The reaction solution was analyzed by SDS–15%PAGE. Finally, each processed mutant CCN′ was further purified by cation chromatography.

### 3.7. Ellman’s Reagent (DTNB) Assay of the proCCN′ Mutant Proteins

The proCCN′ mutant proteins were examined by the Ellman test to confirm the disulfide bond formation. The free thiol content was determined using DTNB. The protein solution (15 µL) was mixed with 15 µL of 10 mM DTNB in 50 mM phosphate buffer (pH 7.0) containing 20 mM NaCl and incubated at 37 °C for 10 min. The absorbance was measured at 405 nm using a GloMax Discover Microplate Reader (Promega Corporation, Madison, WI, USA).

### 3.8. Casein Zymography

Protease activities of the recombinant proteins were assessed by casein zymography [22,42]. Briefly, the mutant proteins purified by cation chromatography were resolved on an SDS-substrate 15% polyacrylamide gel containing casein (1 mg/mL). Samples were mixed with an equal volume of SDS sample buffer (50 mM Tris/HCl, 4.5% SDS, 9% glycerol, 0.2% bromophenol blue, pH 7.0), without boiling, and electrophoresed at 4 °C. Following electrophoresis, SDS was removed by gently agitating the gels twice in 2.5% Triton X-100 for 30 min, followed by another 90 min shaking in 50 mM Tris/HCl (pH 8.0) for the enzyme reaction at 25 °C. The gels were then stained with coomassie blue R250 (60 mg/L) in 10% 2-propanol with 10% acetic acid.

### 3.9. Determination of Enzyme Activity Using Bz-Arg-OEt

The protease activity of the recombinant enzymes was determined using a previously reported method [24,43]. Briefly, enzymatic reactions were performed in 50 mM Tris/HCl (1.0 mL, pH 8.0) containing 25 mM NaCl and 1 mM EDTA as follows: The purified enzyme (approximately 1 μmol/10 μL) was added to 990 μL of Bz-Arg-OEt (final concentration: 25–100 μM) and then incubated at 25 °C for 5 min. Absorbance at 253 nm was recorded every 5 s for a period of 5 min. Initial velocities were plotted against substrate concentrations, and the apparent kinetic parameters, *K*_m_ and *k*_cat_, were obtained by fitting the experimental points to a Lineweaver–Burk plot [44].

### 3.10. Reversed-Phase High-Performance Liquid Chromatography (RP-HPLC)

HPLC analysis was performed to identify the recombinant protein by mass spectrometry following separation. The HPLC apparatus comprised a HITACHI ELITE LaChrom system (L2130) (Hitachi High-Tech Corporation, Tokyo, Japan) equipped with a Hitachi L-3000 detector and a D-2500 chromato-integrator (Hitachi High-Tech Corporation, Tokyo, Japan). The proteins were separated by RP-HPLC using a TSKgel Protein C_4_-300 column (4.6 × 150 mm, Tosoh Co., Ltd., Tokyo, Japan) and confirmed by MALDI-TOF/MS analyses [23,45,46].

### 3.11. Matrix-Assisted Laser Desorption/Ionization Time of Flight Mass Spectrometry (MALDI-TOF/MS)

Molecular masses of the proteins were measured using an MALDI-7090 (SHIMADZU Co., Ltd., Kyoto, Japan) operated in the positive ion mode [23,45,46]. Mass spectrometric analyses were performed in the linear modes using 3,5-dimethoxy-4-hydroxycinnamic acid (Tokyo Chemical Industry Co., Ltd., Tokyo, Japan) as the matrix. For a typical analysis, the lyophilized sample (ca. 0.1 nmol) was dissolved in 0.05% TFA aq/50% CH_3_CN (1 μL), mixed with 1 μL of matrix solution (10 mg/mL), and air-dried on the sample plate prior to MALDI-TOF/MS measurements.

### 3.12. Circular Dichroism (CD) Measurement

CD spectra were obtained using a JASCO J720 spectrometer (JASCO Corporation, Tokyo, Japan) at 25 °C. Proteins purified by cation exchange chromatography were dialyzed against 20 mM sodium phosphate buffer (pH 7.0) containing 0.1 M NaCl and protein concentrations were determined by UV absorbance, as described previously [41]. CD measurements were carried out at 25 °C using a 1 mm path-length cuvette. Spectra were recorded in the 200–280 nm region with a data interval of 0.2 nm, sensitivity of 100 mdeg, response time of 2 s, scanning speed of 10 nm/min, bandwidth of 1 nm, and 4 accumulations [47].

### 3.13. Fluorescence Measurements

Fluorescence measurements were carried out on a Fluorescence Spectrophotometer F-7100 (Hitachi High-Tech Corporation, Tokyo, Japan). For intrinsic tryptophan fluorescence, the excitation wavelength was set at 290 nm and the emission spectra were recorded in the range of 300–400 nm. Each measurement consisted of four consecutive scans, and the averaged spectrum was used for analysis.

For the GdnHCl denaturation experiments, the protein solutions (0.15–0.45 mg/mL) were mixed with 20 mM phosphate buffer (pH 7.0) containing 0.1 M NaCl and various concentrations of GdnHCl to yield final denaturant concentrations of up to 7 M. The mixtures were allowed to stand for 30 min prior to measurement. The degree of unfolding was assessed by tracking the shift in the emission maximum of the intrinsic tryptophan fluorescence spectra. The free energy change for unfolding (Δ*G*°) was calculated from the equilibrium unfolding curves according to the equation Δ*G*° = −RTln(U/N), where U and N represent the concentrations of unfolded (U) and native (N) species at equilibrium, respectively, R is the gas constant, and T is the absolute temperature. The dependence of Δ*G*° on the denaturant concentration was fitted linearly, and the Δ*G*° values were obtained by extrapolation to zero denaturant concentration [34,48]. The protein concentrations were determined by UV absorbance (280 nm), as previously reported [41]. The experiments were performed in triplicate, and the average values are shown.

### 3.14. Molecular Modeling of the CCN′ Mutant Proteins

To obtain the coordinates of the wild-type and mutant proteins, protein modeling was performed using the AlphaFold2 program (ColabFold version 1.5.5) [28]. Then, the areas and volumes of the substrate-binding regions were calculated using the CASTp program (version 3.0) [22,35]. The final molecular structures were depicted using the programs CASTp or PyMOL (version 3.0.3).

## 4. Conclusions

This study elucidated how the introduction of specific disulfide bonds contributed to the structure and function of trypsin-like serine proteases. By introducing two additional disulfide bonds (SS4 and SS5) into cocoonase, a naturally occurring three-disulfide trypsin-like protease, we demonstrated that each disulfide bond plays a distinct and complementary role: SS4 (Cys19–Cys154) reinforces structural rigidity and enhances substrate recognition (*K*_m_), whereas SS5 (Cys134–Cys199) increases catalytic turnover (*k*_cat_) with a thermodynamical stabilizing effect on the hydrophobic core of molecules. The synergistic combination of these two disulfide bonds produced the highest catalytic efficiency among all the mutant proteins, indicating an evolutionary trade-off between activity and conformational robustness.

These results suggest that trypsin-like proteases evolved through a stepwise process in which SS5 was acquired first to enhance both catalytic turnover and structural stability, followed by the later incorporation of SS4 to refine structural rigidity and substrate recognition. Beyond the evolutionary context, this study provides a framework for modulating the balance between the stability and activity of serine proteases through targeted disulfide engineering.

## Figures and Tables

**Table 1:** Kinetic parameters (*K*_m_ and *k*_cat_) of the mature CCN′ mutant proteins measured using BAEE as the substrate.

	*K*_m_ (mol/L)	*k*_cat_ (/s)	*k*_cat_/*K*_m_ (/M·s)
CCN′	1.02 × 10^−3^	0.51 × 10^2^	4.95 × 10^4^
CCN′(SS4,SS5)	3.10 × 10^−4^	2.71 × 10^2^	7.84 × 10^5^
CCN′(SS4)	4.88 × 10^−4^	0.81 × 10^2^	1.87 × 10^5^
CCN′(SS5)	2.92 × 10^−3^	4.75 × 10^2^	1.62 × 10^5^
human trypsin [32]	12.0 × 10^−6^	0.58 × 10^2^	4.83 × 10^6^

**Table 2:** Denaturation midpoint (*C*_m_ value) and unfolding free energy change (Δ*G*°_N→U_) obtained from the fluorescence measurements.

	*C*_m_ (M)	Δ*G*°_N→U_ (kcal/mol)
proCCN′	1.9	3.66
proCCN′(SS4,SS5)	2.4	3.84
proCCN′(SS4)	4.4	3.82
proCCN′(SS5)	1.1	4.55

**Table 3:** Calculated area and volume of the substrate binding pocket of the CCN′ mutant proteins.

	Area (Å^2^)	Volume (Å^3^)
human trypsin [32]	70.5	29.7
CCN	76.6	33.7
CCN′	75.5	33.4
CCN′(SS4,SS5)	74.8	32.2
CCN′(SS4)	74.7	32.0
CCN′(SS5)	75.8	33.5

The characteristic negative volumes were calculated using the CASTp program. CCN and CCN′ represent the wild type and degradation-suppressed cocoonase [24].

## Data Availability

The original contributions presented in this study are included in the article/Supplementary Material. Further inquiries can be directed to the corresponding authors.

## Supplementary Materials

The following supporting information can be downloaded at: https://www.mdpi.com/article/10.3390/molecules31020351/s1, Figure S1: Ellman’s assay evaluating free thiols in the proCCN′ mutant proteins before (left bar) and after (right bar) the refolding reaction. Ellman’s assays were conducted in triplicate (n = 3); Figure S2: RP-HPLC profiles of the proCCN′ mutant proteins purified by cation exchange chromatography. The panels a, b, c, and d represent the chromatograms of the proCCN′, proCCN′(SS4,SS5), proCCN′(SS4), and proCCN′(SS5) proteins, respectively. Proteins in a, c, and d were eluted using a linear gradient of solvent B (solvent A: 0.05% aq TFA; solvent B: 0.05% TFA in CH_3_CN; percentages of solvent B: 10% at 0 min, 50% at 40 min). The proCCN′(SS4,SS5) protein was eluted using a linear gradient of solvent B (solvent A: 0.05% aq TFA; solvent B: 0.05% TFA in CH_3_CN; percentages of solvent B: 15% at 0 min, 55% at 40 min); Figure S3: Synthetic cDNA sequence of the degradation-suppressed mutant protein, proCCN′(SS4,SS5). Codons and corresponding amino acid residues introduced as cysteines are highlighted in blue; Table S1: Mass values of the proCCN′ mutant proteins; Table S2: Primer sequences used for PCR in this study.

## Abbreviations

The following abbreviations are used in this manuscript:

BAEE	Benzoyl-arginine ethyl ester
CCN	cocoonase
CCN′	[K63G,K131G,K133A]-CCN
DPAET	2-(diisopropylamino)ethanethiol
DTNB	5,5′-Dithiobis(2-nitrobenzoic acid)
GSSG	oxidized forms of glutathione
MALDI-TOF/MS	matrix-assisted laser desorption/ionization time of flight mass spectrometry
RP-HPLC	reversed-phase high-performance liquid chromatography
TFA	trifluoroacetic acid
