# Supplementary material for: Structural and Catalytic Roles of the Disulfide Bonds Cys19–Cys154 and Cys134–Cys199 in Trypsin-like Proteases: Evolutionary Insights for Disulfide Bond Acquisition
Source: Molecules. 2026 Jan 19;31(2):351. doi: 10.3390/molecules31020351 (PMC12844481; doi:10.3390/molecules31020351)
Supplement: Supplementary file 1 [file molecules-31-00351-s001.zip › molecules-4062523-supplementary.pdf]

Supplementary Materials

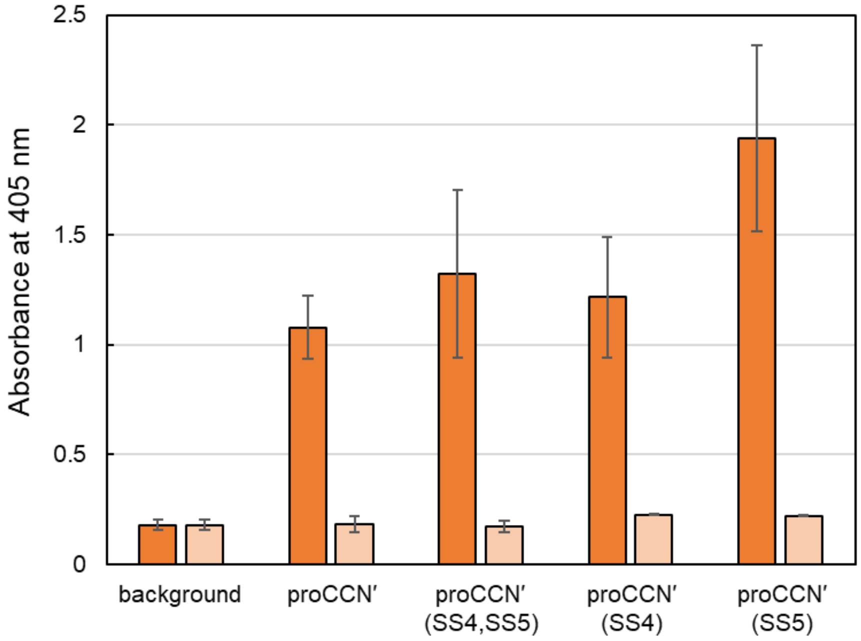

Figure S1. Ellman's assay evaluating free thiols in the proCCN′ mutant proteins before (left bar) and after (right bar) the refolding reaction. Ellman's assays were conducted in triplicate (n = 3).

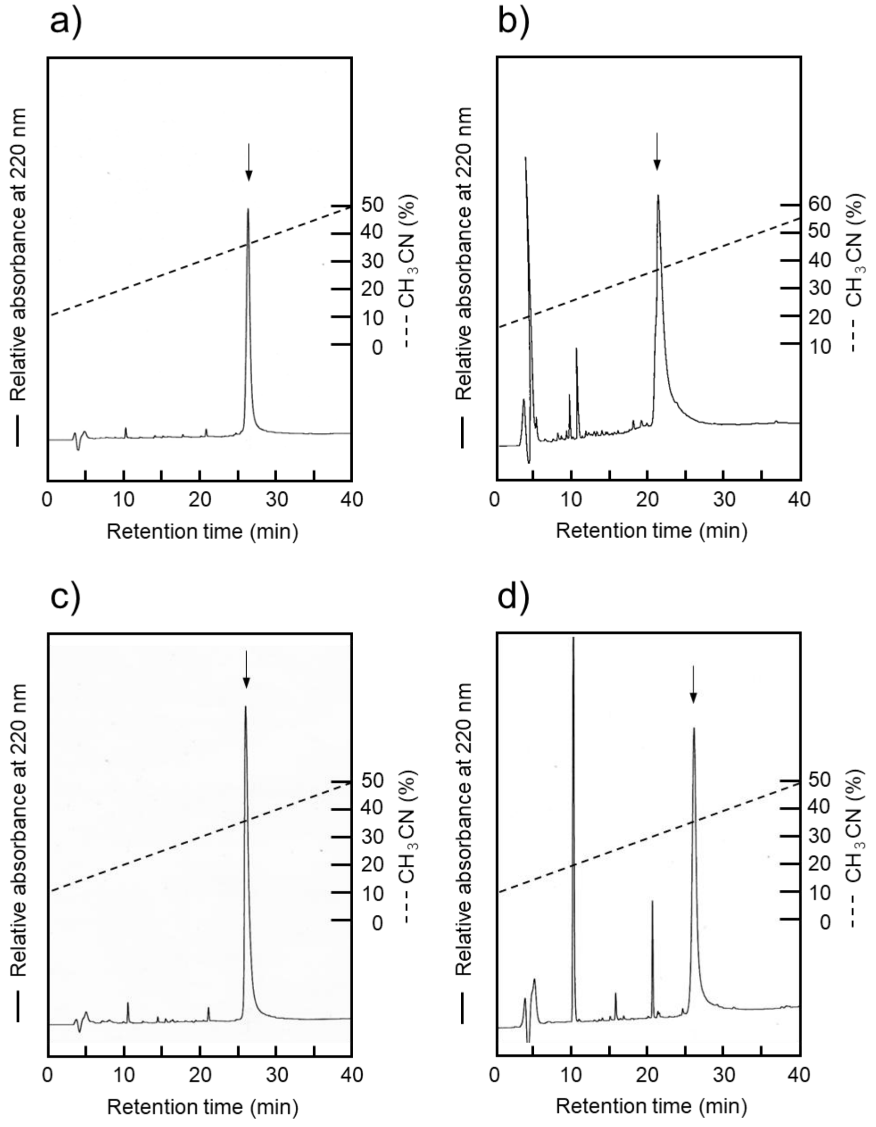

Figure S2. RP-HPLC profiles of the proCCN′ mutant proteins purified by cation exchange chromatography. The panels a, b, c, and d represent the chromatograms of the proCCN′, proCCN′(SS4,SS5), proCCN′(SS4), and proCCN′(SS5) proteins, respectively. Proteins in a, c, and d were eluted using a linear gradient of solvent B (solvent A: 0.05% aq TFA; solvent B: 0.05% TFA in CH_3CN; percentages of solvent B: 10% at 0 min, 50% at 40 min). The proCCN′(SS4,SS5) protein was eluted using a linear gradient of solvent B (solvent A: 0.05% aq TFA; solvent B: 0.05% TFA in CH_3CN; percentages of solvent B: 15% at 0 min, 55% at 40 min). The arrows represent the target proteins.

```
                9           18          27          36          45          54
5'  ACG GAT TCA GAA GCC CTG AGC GAC GAC GAG GAA AAG ATC GTT GGT GGT GAA GAA
    --- --- --- --- --- --- --- --- --- --- --- --- --- --- --- --- --- ---
    Thr Asp Ser Glu Ala Leu Ser Asp Asp Glu Glu Lys Ile Val Gly Gly Glu Glu

                63          72          81          90          99         108
    TGC TCG ATC AAC AAA GTG CCG TAT CAG GCC TAT CTG CTC CTG CAG AAA GAC AAT
    --- --- --- --- --- --- --- --- --- --- --- --- --- --- --- --- --- ---
    Cys Ser Ile Asn Lys Val Pro Tyr Gln Ala Tyr Leu Leu Leu Gln Lys Asp Asn

               117         126         135         144         153         162
    GAG TAC TTC CAG TGT GGC GGT TCA ATC ATT TCT AAA CGC CAC ATT CTG ACT GCG
    --- --- --- --- --- --- --- --- --- --- --- --- --- --- --- --- --- ---
    Glu Tyr Phe Gln Cys Gly Gly Ser Ile Ile Ser Lys Arg His Ile Leu Thr Ala

               171         180         189         198         207         216
    GCA CAT TGC ATT GAG GGT ATC AGC GGT GTG ACA GTA CGC ATT GGG TCT AGC AAT
    --- --- --- --- --- --- --- --- --- --- --- --- --- --- --- --- --- ---
    Ala His Cys Ile Glu Gly Ile Ser Gly Val Thr Val Arg Ile Gly Ser Ser Asn

               225         234         243         252         261         270
    TCC AAC AAA GGC GGC ACG GTC TAT ACC GCG AAA TCC AAG GTT GCC CAT CCG AAA
    --- --- --- --- --- --- --- --- --- --- --- --- --- --- --- --- --- ---
    Ser Asn Lys Gly Gly Thr Val Tyr Thr Ala Lys Ser Lys Val Ala His Pro Lys

               279         288         297         306         315         324
    TAC AAC AGC AAA ACC AAG AAC AAC GAT TTT GCG ATT GTC ACA GTG AAC AAA GAT
    --- --- --- --- --- --- --- --- --- --- --- --- --- --- --- --- --- ---
    Tyr Asn Ser Lys Thr Lys Asn Asn Asp Phe Ala Ile Val Thr Val Asn Lys Asp

               333         342         351         360         369         378
    ATG GCG ATT GAT GGC AAG ACG ACC AAA ATC ATT ACC CTG GCG AAA GAA GGC AGC
    --- --- --- --- --- --- --- --- --- --- --- --- --- --- --- --- --- ---
    Met Ala Ile Asp Gly Lys Thr Thr Lys Ile Ile Thr Leu Ala Lys Glu Gly Ser

               387         396         405         414         423         432
    TCA GTT CCC GAT GGT ACG GCA TGC CTT GTG AGT GGA TGG GGT GCA ACC TCT GAA
    --- --- --- --- --- --- --- --- --- --- --- --- --- --- --- --- --- ---
    Ser Val Pro Asp Gly Thr Ala Cys Leu Val Ser Gly Trp Gly Ala Thr Ser Glu

               441         450         459         468         477         486
    GGA GGT AGC TCG TCC ACC ACT TTG CGT TGC GTT CAT GTC CAA GCA CAC AGT GAC
    --- --- --- --- --- --- --- --- --- --- --- --- --- --- --- --- --- ---
    Gly Gly Ser Ser Ser Thr Thr Leu Arg Cys Val His Val Gln Ala His Ser Asp

               495         504         513         522         531         540
    GAC GAA TGC AAG AAA TAC TTT CGC TCG CTG ACC TCG AAT ATG TTC TGT GCT GGA
    --- --- --- --- --- --- --- --- --- --- --- --- --- --- --- --- --- ---
    Asp Glu Cys Lys Lys Tyr Phe Arg Ser Leu Thr Ser Asn Met Phe Cys Ala Gly

               549         558         567         576         585         594
    CCG CCT GAA GGC GGG AAA GAT AGC TGC CAA GGT GAT AGT GGT GGC CCA GCC GTA
    --- --- --- --- --- --- --- --- --- --- --- --- --- --- --- --- --- ---
    Pro Pro Glu Gly Gly Lys Asp Ser Cys Gln Gly Asp Ser Gly Gly Pro Ala Val

               603         512         621         630         639         648
    TGT GGG AAT GTC CAG TTA GGC GTG GTG TCC TTT GGT GTA GGC TGT GCG CGT AAG
    --- --- --- --- --- --- --- --- --- --- --- --- --- --- --- --- --- ---
    Cys Gly Asn Val Gln Leu Gly Val Val Ser Phe Gly Val Gly Cys Ala Arg Lys

               657         666         675         684         693         702
    AAC AAT CCG GGC ATT TAT GCC AAA GTG AGT GCT GCG GCA AAA TGG ATC AAA TCT
    --- --- --- --- --- --- --- --- --- --- --- --- --- --- --- --- --- ---
    Asn Asn Pro Gly Ile Tyr Ala Lys Val Ser Ala Ala Ala Lys Trp Ile Lys Ser

               711
    ACT GCT GGG TTA TAA   3'
    --- --- --- --- ---
    Thr Ala Gly Leu *
```

Figure S3. Synthetic cDNA sequence of the degradation-suppressed mutant protein, proCCN′(SS4, SS5). Codons and corresponding amino acid residues introduced as cysteines are highlighted in blue.

Table S1. Mass values of the proCCN′ mutant proteins.

mutant proteins	$[M+H]^{+}_{calculated}$	$[M+H]^{+}_{observed}$
proCCN'	24992 Da	24972 Da
proCCN'(SS4,SS5)	24975 Da	25004 Da
proCCN'(SS4)	25012 Da	25005 Da
proCCN'(SS5)	24955 Da	24926 Da

Table S2. Primer sequences used for PCR in this study.

Primer name	Primer direction	Primer sequence	Mutation
K8D sense	forward	5′ CTGTCAGACGACGAAGAGAAGATTGTC 3′	Lys8Asp
K8D anti	reverse	5′ TTCGTCGTCTGACAGCGCTTCAGAAT 3′	Lys8Asp
[C19I]-proCCN′	forward	5′ GAAGAAATCTCGATCAACAAAGTG 3′	Cys19Ile
[C19I]-proCCN′	reverse	5′ GATCGAGATTTCTTCACCACCAACG 3′	Cys19Ile
[C154A]-proCCN′	forward	5′ TTGCGTGCTGTTCATGTCCAAGCAC 3′	Cys154Ala
[C154A]-proCCN′	reverse	5′ ATGAACAGCACGCAAAGTGGTGGA 3′	Cys154Ala
[C134L]-proCCN′	forward	5′ ACGGCACTGCTTGTGAGTGGATGGG 3′	Cys134Leu
[C134L]-proCCN′	reverse	5′ CACAAGCAGTGCCGTACCATCGGG 3′	Cys134Leu
[C199K]-proCCN′	forward	5′ GCCGTAAAAGGGAATGTCCAGTTAG 3′	Cys199Lys
[C199K]-proCCN′	reverse	5′ ATTCCCTTTTACGGCTGGGCCACC 3′	Cys199Lys
T7 promoter	forward	5′ TAATACGACTCACTATAGG 3′	-
T7 terminater	reverse	5′ CCCAAGGGGTTATGCTA 3′	-
